# {2,6-Bis[(2,6-diisopropyl­phosphan­yl)­oxy]-4-fluoro­phenyl-κ^3^ *P*,*C* ^1^,*P*′}(1*H*-pyrazole-κ*N* ^2^)nickel(II) hexa­fluoro­phosphate

**DOI:** 10.1107/S1600536812039207

**Published:** 2012-09-19

**Authors:** Man-Lung Kwan, Sara J. Conry, Charles S. Carfagna, Loren P. Press, Oleg V. Ozerov, Norris W. Hoffman, Richard E. Sykora

**Affiliations:** a Department of Chemistry, John Carroll University, University Heights, OH 44118, USA; b Department of Chemistry, Texas A&M University, College Station, TX 77843, USA; c Department of Chemistry, University of South Alabama, Mobile, AL 36688, USA

## Abstract

The title compound, [Ni(C_18_H_30_FO_2_P_2_)(C_3_H_4_N_2_)]PF_6_, was prepared by halide abstraction with TlPF_6_ in the presence of CH_3_CN in CDCl_3_ from the respective neutral pincer chlorido analogue followed by addition of pyrazole. The PO—C—OP pincer ligand acts in typical *trans*-P_2_ tridentate fashion to generate a distorted square-planar nickel structure. The Ni—N(pyrazole) distance is 1.925 (2) Å and the plane of the pyrazole ligand is rotated 56.2 (1)° relative to the approximate square plane surrounding the Ni^II^ center in which the pyrazole is bound to the Ni^II^ atom through its *sp* ^2^-hybridized N atom. This Ni—N distance is similar to bond lengths in the other reported Ni^II^ pincer-ligand square-planar pyrazole complex structures; however, its dihedral angle is significantly larger than any of those for the latter set of pyrazole complexes.

## Related literature

For recent studies on the chemistry of *d*-block PO—C—OP pincer complexes, see Chen *et al.* (2012 ▶); Zhang *et al.* (2012 ▶); Salah & Zargarian (2011 ▶); Hoffman *et al.* (2009 ▶); Wicker *et al.* (2011 ▶). For structures of other Ni^II^ pincer-ligand square-planar pyrazole complexes, see Salem *et al.* (2007 ▶, 2008 ▶); Peng *et al.* (2010 ▶). For information regarding the ^19^F NMR reference, see: Ji *et al.* (2005 ▶).
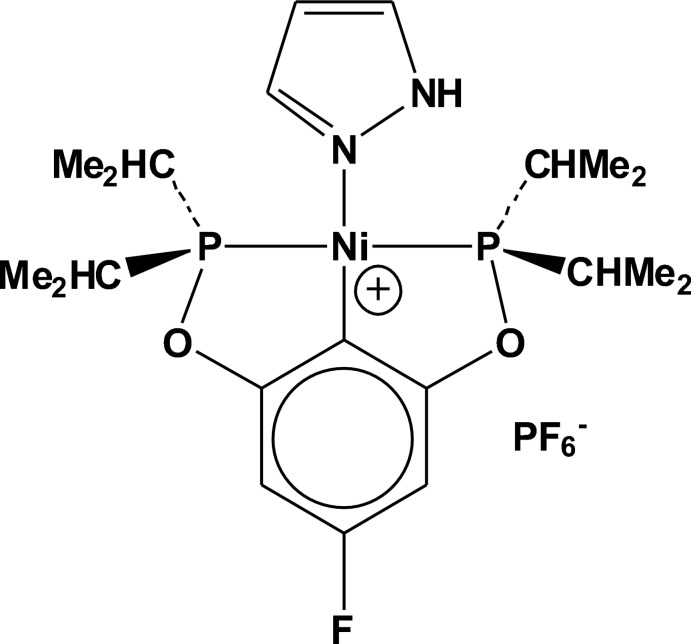


## Experimental

### 

#### Crystal data

[Ni(C_18_H_30_FO_2_P_2_)(C_3_H_4_N_2_)]PF_6_
*M* *_r_* = 631.12
Monoclinic, 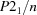

*a* = 9.0380 (9) Å
*b* = 20.1878 (16) Å
*c* = 16.1480 (16) Å
β = 98.659 (8)°
*V* = 2912.7 (5) Å^3^
*Z* = 4
Mo *K*α radiation
μ = 0.90 mm^−1^
*T* = 290 K
0.58 × 0.52 × 0.34 mm

#### Data collection

Enraf–Nonius CAD-4 diffractometer
Absorption correction: ψ scan (North *et al.*, 1968 ▶) *T*_min_ = 0.265, *T*_max_ = 0.315
5464 measured reflections
5122 independent reflections
3432 reflections with *I* > 2σ(*I*)
*R*_int_ = 0.024
3 standard reflections every 120 min intensity decay: none

#### Refinement

*R*[*F* ^2^ > 2σ(*F* ^2^)] = 0.037
*wR*(*F* ^2^) = 0.100
*S* = 1.00
5122 reflections
325 parameters
H-atom parameters constrained
Δρ_max_ = 0.22 e Å^−3^
Δρ_min_ = −0.29 e Å^−3^

### 

Data collection: *CAD-4-PC* (Enraf–Nonius, 1993 ▶); cell refinement: *CAD-4-PC*; data reduction: *XCAD4-PC* (Harms & Wocadlo, 1995) ▶; program(s) used to solve structure: *SHELXS97* (Sheldrick, 2008 ▶); program(s) used to refine structure: *SHELXL97* (Sheldrick, 2008 ▶); molecular graphics: *OLEX2* (Dolomanov *et al.*, 2009 ▶); software used to prepare material for publication: *publCIF* (Westrip, 2010 ▶).

## Supplementary Material

Crystal structure: contains datablock(s) I, global. DOI: 10.1107/S1600536812039207/hg5249sup1.cif

Structure factors: contains datablock(s) I. DOI: 10.1107/S1600536812039207/hg5249Isup2.hkl

Additional supplementary materials:  crystallographic information; 3D view; checkCIF report

## Figures and Tables

**Fig. 1.**
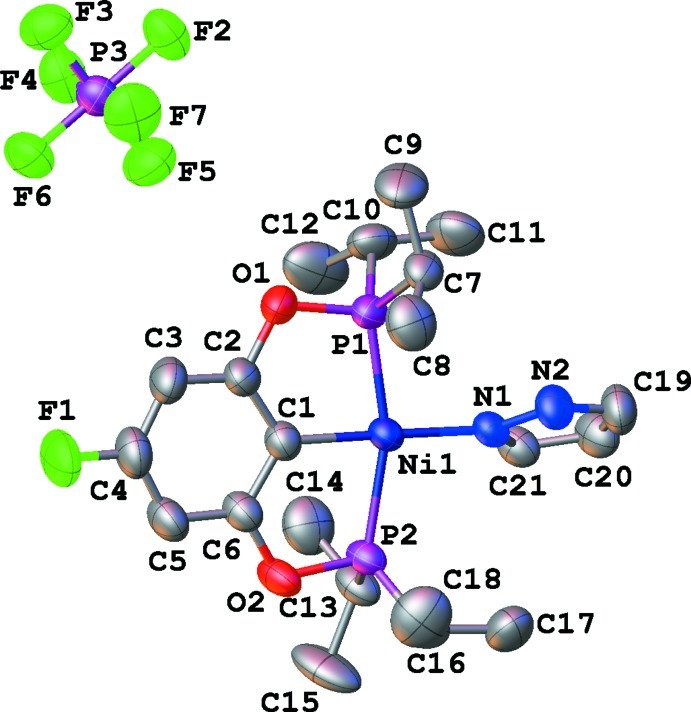
A thermal ellipsoid plot (50%) of the title compound showing the labeling scheme.

**Fig. 2.**
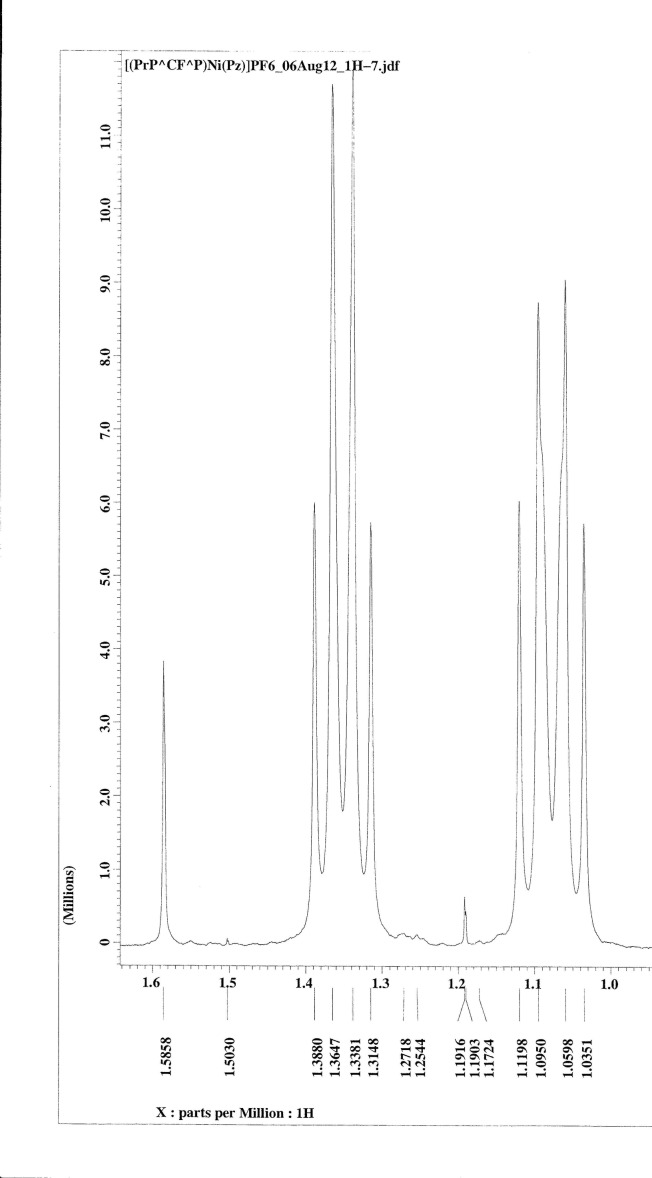
1H NMR spectrum of the title compound at 23 °C.

**Fig. 3.**
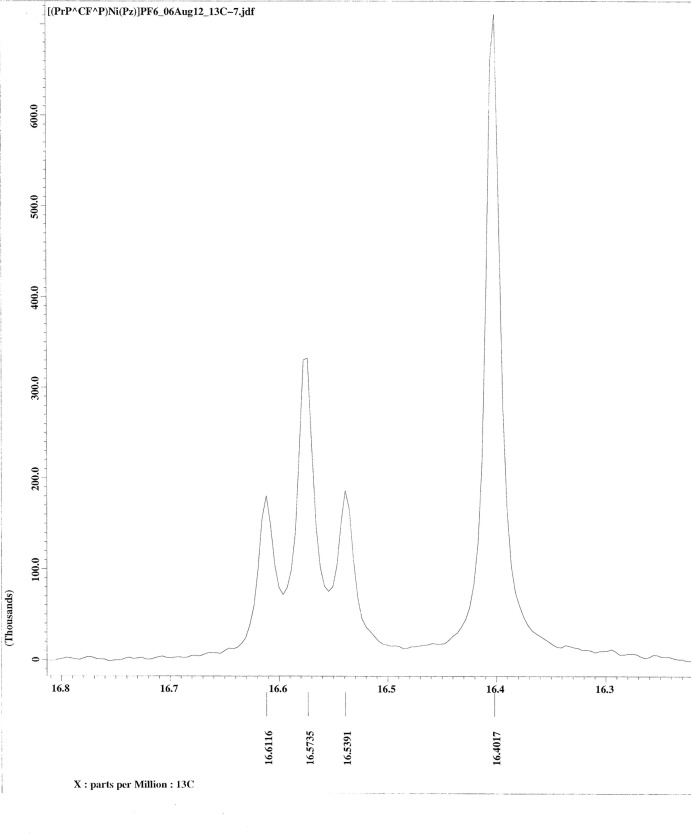
13C NMR spectrum of the title compound at 23 °C.

## supplementary crystallographic information

### Comment

Considerable attention has recently been devoted to nickel PO—C—OP pincer complexes (*e.g.*, Salah & Zargarian, 2011; Chen *et al.*, 2012; Zhang *et al.*, 2012). Our interest in studying relative binding affinities of metal centers for ligands of moderate donor power using ^19^F and ^31^P NMR spectroscopy (Hoffman *et al.*, 2009) to monitor ligand-substitution equilibria led us to prepare the title complex (I). The fluoro-pincer ligand precursor was generated by heating 5-fluororesorcinol and diisopropylchlorophosphine in THF in the presence of triethylamine, and then anhydrous NiCl_2_ was added to form the (PO—C—OP)NiCl complex. Chloride abstraction with TlPF_6_ in the presence of CH_3_CN from this species followed by addition of pyrazole afforded an excellent yield of the cationic complex whose structure is shown below in Fig 1. Suitable single crystals were grown *via* vapor diffusion of methyl *tert*-butyl ether into a CDCl_3_ solution of the highly soluble reaction product at room temperature. Its Ni—N distance, 1.925 (2) Å, fell within the range of such values for the five square-planar Ni^II^-pincer unsubstituted-pyrazole complexes found in the Cambridge Structural Database (Salem *et al.* (2008), Peng *et al.* (2010), and Salem *et al.* (2007)). However, its pyrazole-ring/Ni-coordination-plane dihedral angle, 56.2 (1)°, falls significantly outside the range (3–28°) of those for the latter set of pyrazole complexes, in which none of the pendant-ligand arms exert meaningful steric force upon the pyrazole position. The C—F bond length for this complex, 1.355 (4) Å, is identical within experimental error to that, 1.357 (3) Å, of Pd{(3–2,6-[(C_6_H_5_)_2_PO]_2_-C_6_H_2_-4-F}(C_4_H_4_NO_4_S), a Pd(II) acesulfamato complex containing a similar fluoro-pincer ligand (Wicker *et al.*, 2011). Detailed lists of dimensions are available in the archived CIF.

### Experimental

The first step entailed generating a neutral nickel(II) halido pincer complex, abbreviated as Pr{Ni_F_}Cl. An anhydrous solution of 5-fluororesorcinol (330 mg, 2.58 mmol) in THF was prepared in a 100-ml Kontes Teflon screw-cap flask inside a glovebox. To this solution was added *via* syringe 1.50 ml NEt_3_ (10.77 mmol) followed by 850 µ*L* of ClPPr^i^_2_ (5.34 mmol); immediately a large volume of white precipitate formed. The flask was brought out of the glovebox and heated for 20 minutes at 80 °C; thereafter the flask was returned to the glovebox, where 0.57 grams of anhydrous NiCl_2_ (4.40 mmol) was added to afford an orange mixture. After 24 h under heavy stirring at 80 °C, the resulting green-yellow mixture was filtered through a plug of Celite and concentrated under vacuum. This solution was layered with pentane and left overnight in a -30 °C freezer to give dark yellow-brown crystals of Pr{Ni_F_}Cl (608 mg, 52% yield).

Complex I, [Ni(C_3_H_2_N_2_)(C_12_H_16_FO_2_P_2_)]PF_6_, abbreviated as Pr{Ni_F_}(Pz)]PF_6_, was prepared as follows. Pr{Ni_F_}Cl (32.4 mg, 0.0799 mmol) was stirred at ambient temperature for 24 h with TlPF_6_ (Strem Chemicals; 1.15 mol equiv.) in CDCl_3_ (Cambridge Isotopes Lab; 1.25 ml) to which 25 µ*L* CH_3_CN (Fisher reagent) had been added. The resulting mixture was filtered to remove insoluble TlCl and excess TlPF_6_. To the resulting yellow-orange solution of Pr{Ni_F_}(CH_3_CN)]PF_6_ was added a slight excess of solid pyrazole (Aldrich; 5.7 mg), and the reaction solution was stirred one hour. Then it was subjected to vapor diffusion with 30 ml me thyl *tert*-butyl ether (MTBE; Fisher reagent) at 22 °C for 3 days. The very pale yellow liquid of the resulting mixture was removed from the vial by a small-diameter syringe needle, and the rod-like orange crystals were washed twice with 1.5 ml of MTBE, removed from the vial, and then air-dried overnight in the dark (91% yield). The complex was characterized by NMR at 24 °C in CDCl_3_. Unusual features of the spectra are discussed below the data tabulation.

^1^H: (relative to internal TMS) δ 11.66 (broad singlet, 0.74 H); δ 8.07 (overlapping doublet of doublets, 1H); δ 7.64 (overlapping doublet of doublets, 1H); δ 6.58 (2*H*, doublet, ^3^J_F—H_=9.6 Hz) δ 2.34 (4*H*, septuplet, ^3^J_H—H_=7.1 Hz); δ 1.35 (overlapping inequivalent triplets, 12H); δ 1.08 (overlapping inequivalent triplets, 12H)

^19^F: (relative to internal C_6_H_5_CF_3_ at δ -63.00; (Ji *et al.*, 2005)) aryl-F at δ -109.41 (triplet of triplets, ^3^J_H—F_=9.6 Hz, ^5^J_P—F_=1.0 Hz) and counterion PF_6_^-^ at δ -72.28 (doublet, ^2^J_P—F_=713 Hz)

^31^P: (relative to external 85% aq. phosphoric acid) pincer-P at δ 190.31 (doublet, ^5^J_P—F_=1.0 Hz) and counterion PF_6_^-^ at δ -143.79 (septuplet, ^2^J_P—F_=713 Hz)

^13^C: (relative to internal TMS)

Pyrazole: δ 108.59(*s*), δ 135.21(*s*), δ 141.43(*s*).

Pincer Aryl: *ipso*δ 115.49(t of d; ^4^J_F—C_=2.9 Hz, ^2^J_P—C_=21 Hz) *ortho*δ 168.55 (d of t; ^3^J_F—C_=15 Hz, ^2^J_P—C_~7.4 Hz) *meta*δ 94.89(d of t; ^2^J_F—C_=33 Hz, ^4^J_P—C_~6.6 Hz) *para*δ 165.16 (d; ^1^J_F—C_=246 Hz Pr^i^_2_P: methyne, δ 27.66(t; ^1^J_P—C_=11 Hz) methyls δ 16.58(t; ^2^J_P—C_=11 Hz), δ 16.41(*s*)

Integrals recorded for ^1^H signals of Pr{Ni_F_}(Pz)]PF_6_ match expected values except for the low-field pyrazole N—H resonance in which strong hydrogen-bonding to the hexafluorophosphate counterion is likely. Overlap of ^1^H triplets for the inequivalent isopropyl methyl groups created by different rotation rates around the Ni—Pz, P—C, and C—C bonds affords pseudo-quartets centered at δ 1.35 and δ 1.08 (Fig. 2). The same rotational phenomena generate a more unusual ^13^C-NMR methyls pattern (Fig. 3), in which a triplet at δ 16.58 (^2^J_P—C_=11 Hz) and singlet at δ 16.41 (no measurable P—C coupling observed) of equal intensity appear. Recording the ^13^C-NMR spectrum with longer relaxation delay (4 s *versus* 1 s) or at 44 °C affords no change in this pattern.

### Refinement

Hydrogen atoms were placed in calculated positions and allowed to ride during subsequent refinement, with *U*_iso_(H) = 1.2*U*_eq_(C) and C—H distances of 0.93 Å for the H atoms on the pyrazole carbons, *U*_iso_(H) = 1.2*U*_eq_(N) and an N—H distance of 0.86 Å for the H atom on the pyrazole N, *U*_iso_(H) = 1.2*U*_eq_(C) and C—H distances of 0.98 Å for the H atoms on the tertiary carbons, and *U*_iso_(H) = 1.5*U*_eq_(C) and C—H distances of 0.96 Å for the methyl H atoms.

### Crystal data

**Table:**

[Ni(C_18_H_30_FO_2_P_2_)(C_3_H_4_N_2_)]PF_6_	*F*(000) = 1304
*M**_r_* = 631.12	*D*_x_ = 1.439 Mg m^−^^3^
Monoclinic, *P*2_1_/*n*	Mo *K*α radiation, λ = 0.71073 Å
Hall symbol: -P 2yn	Cell parameters from 25 reflections
*a* = 9.0380 (9) Å	θ = 9.5–13.0°
*b* = 20.1878 (16) Å	µ = 0.90 mm^−^^1^
*c* = 16.1480 (16) Å	*T* = 290 K
β = 98.659 (8)°	Prism, yellow
*V* = 2912.7 (5) Å^3^	0.58 × 0.52 × 0.34 mm
*Z* = 4	

### Data collection

**Table:**

Enraf–Nonius CAD-4 diffractometer	3432 reflections with *I* > 2σ(*I*)
Radiation source: fine-focus sealed tube	*R*_int_ = 0.024
Graphite monochromator	θ_max_ = 25.0°, θ_min_ = 2.0°
θ/2θ scans	*h* = 0→10
Absorption correction: ψ scan (North *et al.*, 1968)	*k* = 0→23
*T*_min_ = 0.265, *T*_max_ = 0.315	*l* = −19→18
5464 measured reflections	3 standard reflections every 120 min
5122 independent reflections	intensity decay: none

### Refinement

**Table:**

Refinement on *F*^2^	Primary atom site location: structure-invariant direct methods
Least-squares matrix: full	Secondary atom site location: difference Fourier map
*R*[*F*^2^ > 2σ(*F*^2^)] = 0.037	Hydrogen site location: inferred from neighbouring sites
*wR*(*F*^2^) = 0.100	H-atom parameters constrained
*S* = 1.00	*w* = 1/[σ^2^(*F*_o_^2^) + (0.0436*P*)^2^ + 0.9656*P*] where *P* = (*F*_o_^2^ + 2*F*_c_^2^)/3
5122 reflections	(Δ/σ)_max_ < 0.001
325 parameters	Δρ_max_ = 0.22 e Å^−^^3^
0 restraints	Δρ_min_ = −0.29 e Å^−^^3^

### Special details

**Table:**

Geometry. All e.s.d.'s (except the e.s.d. in the dihedral angle between two l.s. planes) are estimated using the full covariance matrix. The cell e.s.d.'s are taken into account individually in the estimation of e.s.d.'s in distances, angles and torsion angles; correlations between e.s.d.'s in cell parameters are only used when they are defined by crystal symmetry. An approximate (isotropic) treatment of cell e.s.d.'s is used for estimating e.s.d.'s involving l.s. planes.
Refinement. Refinement of *F*^2^ against ALL reflections. The weighted *R*-factor *wR* and goodness of fit *S* are based on *F*^2^, conventional *R*-factors *R* are based on *F*, with *F* set to zero for negative *F*^2^. The threshold expression of *F*^2^ > 2σ(*F*^2^) is used only for calculating *R*-factors(gt) *etc*. and is not relevant to the choice of reflections for refinement. *R*-factors based on *F*^2^ are statistically about twice as large as those based on *F*, and *R*- factors based on ALL data will be even larger.

### Fractional atomic coordinates and isotropic or equivalent isotropic displacement parameters (Å^2^)

**Table:**

	*x*	*y*	*z*	*U*_iso_*/*U*_eq_	
Ni1	0.32898 (5)	0.127947 (18)	0.28825 (2)	0.04161 (12)	
P1	0.23694 (10)	0.18930 (4)	0.37905 (6)	0.0479 (2)	
P2	0.39378 (11)	0.04384 (4)	0.21782 (5)	0.0479 (2)	
P3	0.20240 (11)	0.13469 (5)	0.89133 (6)	0.0559 (2)	
O1	0.1773 (3)	0.13751 (11)	0.44521 (15)	0.0617 (7)	
O2	0.3652 (3)	−0.02228 (10)	0.27213 (14)	0.0609 (7)	
N1	0.3831 (3)	0.19614 (12)	0.21502 (16)	0.0446 (6)	
N2	0.4812 (3)	0.24504 (14)	0.23638 (18)	0.0568 (7)	
H2A	0.5305	0.2502	0.2858	0.068*	
F1	0.1762 (3)	−0.08905 (11)	0.51487 (15)	0.0953 (8)	
F2	0.1974 (3)	0.21394 (10)	0.87960 (15)	0.0789 (7)	
F3	0.1327 (3)	0.14307 (11)	0.97561 (13)	0.0792 (7)	
F4	0.0382 (2)	0.13121 (10)	0.84026 (14)	0.0761 (6)	
F5	0.2707 (3)	0.12876 (13)	0.80763 (16)	0.0966 (8)	
F6	0.2071 (3)	0.05746 (10)	0.90361 (15)	0.0837 (7)	
F7	0.3643 (3)	0.14096 (13)	0.94371 (17)	0.0969 (8)	
C1	0.2761 (4)	0.05995 (15)	0.3580 (2)	0.0487 (8)	
C2	0.2103 (4)	0.07201 (16)	0.4286 (2)	0.0519 (8)	
C3	0.1765 (4)	0.02385 (18)	0.4828 (2)	0.0658 (10)	
H3A	0.1341	0.0340	0.5303	0.079*	
C4	0.2087 (5)	−0.04016 (19)	0.4630 (2)	0.0680 (11)	
C5	0.2699 (5)	−0.05769 (17)	0.3935 (2)	0.0650 (10)	
H5A	0.2883	−0.1017	0.3813	0.078*	
C6	0.3028 (4)	−0.00660 (16)	0.3426 (2)	0.0527 (8)	
C7	0.3621 (4)	0.24172 (17)	0.4498 (2)	0.0573 (9)	
H7A	0.4045	0.2751	0.4161	0.069*	
C8	0.4897 (5)	0.1988 (2)	0.4920 (3)	0.0838 (13)	
H8A	0.5581	0.2255	0.5291	0.126*	
H8B	0.5413	0.1794	0.4501	0.126*	
H8C	0.4503	0.1643	0.5233	0.126*	
C9	0.2836 (5)	0.2780 (2)	0.5141 (3)	0.0912 (15)	
H9A	0.3545	0.3054	0.5487	0.137*	
H9B	0.2422	0.2463	0.5485	0.137*	
H9C	0.2047	0.3051	0.4855	0.137*	
C10	0.0709 (4)	0.23715 (19)	0.3406 (3)	0.0684 (10)	
H10A	0.0314	0.2559	0.3888	0.082*	
C11	0.1109 (5)	0.2940 (2)	0.2859 (3)	0.0895 (14)	
H11A	0.0225	0.3190	0.2659	0.134*	
H11B	0.1525	0.2765	0.2392	0.134*	
H11C	0.1831	0.3223	0.3183	0.134*	
C12	−0.0481 (5)	0.1917 (2)	0.2934 (3)	0.1067 (18)	
H12A	−0.1359	0.2171	0.2730	0.160*	
H12B	−0.0734	0.1577	0.3304	0.160*	
H12C	−0.0099	0.1717	0.2470	0.160*	
C13	0.2776 (4)	0.02806 (16)	0.1181 (2)	0.0573 (9)	
H13A	0.3016	0.0619	0.0786	0.069*	
C14	0.1133 (5)	0.0365 (2)	0.1272 (3)	0.0880 (14)	
H14A	0.0522	0.0287	0.0742	0.132*	
H14B	0.0968	0.0808	0.1456	0.132*	
H14C	0.0875	0.0054	0.1677	0.132*	
C15	0.3067 (7)	−0.0397 (2)	0.0809 (3)	0.1052 (18)	
H15A	0.2431	−0.0450	0.0281	0.158*	
H15B	0.2856	−0.0740	0.1187	0.158*	
H15C	0.4095	−0.0425	0.0727	0.158*	
C16	0.5878 (4)	0.03345 (18)	0.2048 (2)	0.0621 (10)	
H16A	0.5979	−0.0099	0.1789	0.074*	
C17	0.6377 (5)	0.0857 (2)	0.1476 (3)	0.0824 (13)	
H17A	0.7405	0.0783	0.1417	0.124*	
H17B	0.6274	0.1288	0.1713	0.124*	
H17C	0.5767	0.0832	0.0936	0.124*	
C18	0.6859 (5)	0.0334 (3)	0.2900 (3)	0.1030 (16)	
H18A	0.7886	0.0275	0.2828	0.155*	
H18B	0.6562	−0.0023	0.3233	0.155*	
H18C	0.6748	0.0747	0.3177	0.155*	
C19	0.4934 (5)	0.28489 (18)	0.1717 (2)	0.0706 (11)	
H19A	0.5543	0.3221	0.1725	0.085*	
C20	0.4012 (5)	0.26112 (18)	0.1053 (2)	0.0665 (10)	
H20A	0.3859	0.2782	0.0512	0.080*	
C21	0.3346 (4)	0.20647 (16)	0.1337 (2)	0.0535 (9)	
H21A	0.2648	0.1800	0.1008	0.064*	

### Atomic displacement parameters (Å^2^)

**Table:**

	*U*^11^	*U*^22^	*U*^33^	*U*^12^	*U*^13^	*U*^23^
Ni1	0.0490 (2)	0.0336 (2)	0.0428 (2)	−0.00062 (18)	0.00846 (17)	−0.00333 (17)
P1	0.0493 (5)	0.0408 (4)	0.0564 (5)	−0.0048 (4)	0.0168 (4)	−0.0095 (4)
P2	0.0663 (6)	0.0357 (4)	0.0420 (5)	0.0042 (4)	0.0095 (4)	−0.0027 (3)
P3	0.0559 (6)	0.0504 (5)	0.0615 (6)	0.0087 (4)	0.0092 (4)	0.0069 (4)
O1	0.0703 (16)	0.0510 (14)	0.0714 (16)	−0.0127 (12)	0.0351 (13)	−0.0105 (12)
O2	0.0977 (19)	0.0355 (12)	0.0512 (14)	0.0088 (12)	0.0169 (13)	0.0017 (10)
N1	0.0494 (16)	0.0355 (14)	0.0489 (16)	−0.0027 (12)	0.0070 (13)	−0.0046 (11)
N2	0.0654 (19)	0.0532 (16)	0.0511 (17)	−0.0163 (15)	0.0069 (14)	−0.0071 (14)
F1	0.145 (2)	0.0653 (15)	0.0830 (16)	−0.0156 (15)	0.0418 (16)	0.0215 (13)
F2	0.0833 (16)	0.0520 (12)	0.0982 (17)	0.0014 (11)	0.0041 (13)	0.0099 (12)
F3	0.0881 (16)	0.0868 (16)	0.0655 (14)	0.0200 (13)	0.0205 (12)	0.0081 (12)
F4	0.0689 (14)	0.0729 (14)	0.0799 (15)	−0.0058 (11)	−0.0105 (11)	0.0113 (12)
F5	0.119 (2)	0.0951 (18)	0.0864 (17)	0.0124 (15)	0.0492 (15)	0.0068 (14)
F6	0.1030 (19)	0.0539 (13)	0.0971 (18)	0.0157 (12)	0.0244 (14)	0.0103 (12)
F7	0.0593 (14)	0.109 (2)	0.116 (2)	0.0090 (14)	−0.0080 (13)	0.0128 (16)
C1	0.055 (2)	0.0428 (17)	0.0487 (19)	−0.0047 (15)	0.0081 (16)	−0.0002 (14)
C2	0.056 (2)	0.0430 (18)	0.058 (2)	−0.0083 (16)	0.0145 (17)	−0.0068 (16)
C3	0.081 (3)	0.063 (2)	0.057 (2)	−0.016 (2)	0.024 (2)	0.0002 (19)
C4	0.086 (3)	0.057 (2)	0.062 (2)	−0.016 (2)	0.014 (2)	0.0118 (19)
C5	0.092 (3)	0.0413 (19)	0.063 (2)	−0.0023 (18)	0.014 (2)	0.0042 (17)
C6	0.070 (2)	0.0414 (17)	0.0461 (19)	−0.0010 (16)	0.0062 (17)	0.0003 (15)
C7	0.064 (2)	0.053 (2)	0.057 (2)	−0.0149 (17)	0.0145 (17)	−0.0149 (17)
C8	0.075 (3)	0.086 (3)	0.085 (3)	−0.015 (2)	−0.006 (2)	−0.004 (2)
C9	0.117 (4)	0.084 (3)	0.079 (3)	−0.018 (3)	0.034 (3)	−0.037 (3)
C10	0.056 (2)	0.067 (2)	0.085 (3)	0.0098 (19)	0.019 (2)	−0.015 (2)
C11	0.092 (3)	0.072 (3)	0.104 (4)	0.032 (3)	0.014 (3)	0.007 (3)
C12	0.058 (3)	0.104 (4)	0.149 (5)	0.007 (3)	−0.013 (3)	−0.032 (3)
C13	0.085 (3)	0.0400 (18)	0.0441 (19)	−0.0001 (18)	0.0004 (18)	−0.0052 (15)
C14	0.082 (3)	0.093 (3)	0.083 (3)	−0.020 (3)	−0.008 (2)	−0.007 (3)
C15	0.187 (5)	0.054 (2)	0.064 (3)	0.021 (3)	−0.013 (3)	−0.025 (2)
C16	0.067 (2)	0.056 (2)	0.064 (2)	0.0121 (19)	0.0124 (19)	−0.0068 (18)
C17	0.073 (3)	0.083 (3)	0.098 (3)	0.009 (2)	0.035 (2)	0.004 (3)
C18	0.080 (3)	0.130 (4)	0.094 (4)	0.011 (3)	−0.003 (3)	0.013 (3)
C19	0.087 (3)	0.056 (2)	0.072 (3)	−0.022 (2)	0.023 (2)	0.004 (2)
C20	0.093 (3)	0.055 (2)	0.051 (2)	0.004 (2)	0.012 (2)	0.0097 (18)
C21	0.066 (2)	0.0458 (19)	0.046 (2)	0.0012 (17)	−0.0004 (17)	−0.0013 (15)

### Geometric parameters (Å, º)

**Table:**

Ni1—C1	1.883 (3)	C8—H8C	0.9600
Ni1—N1	1.925 (2)	C9—H9A	0.9600
Ni1—P2	2.1727 (9)	C9—H9B	0.9600
Ni1—P1	2.1779 (9)	C9—H9C	0.9600
P1—O1	1.643 (2)	C10—C11	1.525 (5)
P1—C10	1.814 (4)	C10—C12	1.528 (5)
P1—C7	1.821 (3)	C10—H10A	0.9800
P2—O2	1.639 (2)	C11—H11A	0.9600
P2—C16	1.810 (4)	C11—H11B	0.9600
P2—C13	1.814 (3)	C11—H11C	0.9600
P3—F6	1.571 (2)	C12—H12A	0.9600
P3—F5	1.573 (2)	C12—H12B	0.9600
P3—F7	1.581 (2)	C12—H12C	0.9600
P3—F4	1.587 (2)	C13—C14	1.523 (5)
P3—F3	1.592 (2)	C13—C15	1.532 (5)
P3—F2	1.611 (2)	C13—H13A	0.9800
O1—C2	1.391 (4)	C14—H14A	0.9600
O2—C6	1.380 (4)	C14—H14B	0.9600
N1—C21	1.337 (4)	C14—H14C	0.9600
N1—N2	1.337 (3)	C15—H15A	0.9600
N2—C19	1.336 (4)	C15—H15B	0.9600
N2—H2A	0.8600	C15—H15C	0.9600
F1—C4	1.355 (4)	C16—C17	1.515 (5)
C1—C2	1.384 (4)	C16—C18	1.521 (5)
C1—C6	1.394 (4)	C16—H16A	0.9800
C2—C3	1.373 (5)	C17—H17A	0.9600
C3—C4	1.373 (5)	C17—H17B	0.9600
C3—H3A	0.9300	C17—H17C	0.9600
C4—C5	1.370 (5)	C18—H18A	0.9600
C5—C6	1.379 (5)	C18—H18B	0.9600
C5—H5A	0.9300	C18—H18C	0.9600
C7—C8	1.519 (5)	C19—C20	1.344 (5)
C7—C9	1.530 (5)	C19—H19A	0.9300
C7—H7A	0.9800	C20—C21	1.369 (5)
C8—H8A	0.9600	C20—H20A	0.9300
C8—H8B	0.9600	C21—H21A	0.9300
			
C1—Ni1—N1	178.79 (12)	H8B—C8—H8C	109.5
C1—Ni1—P2	81.73 (10)	C7—C9—H9A	109.5
N1—Ni1—P2	97.11 (8)	C7—C9—H9B	109.5
C1—Ni1—P1	81.64 (10)	H9A—C9—H9B	109.5
N1—Ni1—P1	99.49 (8)	C7—C9—H9C	109.5
P2—Ni1—P1	163.19 (4)	H9A—C9—H9C	109.5
O1—P1—C10	103.04 (16)	H9B—C9—H9C	109.5
O1—P1—C7	101.28 (15)	C11—C10—C12	111.9 (4)
C10—P1—C7	108.00 (18)	C11—C10—P1	110.0 (3)
O1—P1—Ni1	105.77 (9)	C12—C10—P1	109.6 (3)
C10—P1—Ni1	116.93 (14)	C11—C10—H10A	108.4
C7—P1—Ni1	119.25 (12)	C12—C10—H10A	108.4
O2—P2—C16	101.63 (16)	P1—C10—H10A	108.4
O2—P2—C13	102.43 (15)	C10—C11—H11A	109.5
C16—P2—C13	108.56 (18)	C10—C11—H11B	109.5
O2—P2—Ni1	106.24 (9)	H11A—C11—H11B	109.5
C16—P2—Ni1	119.58 (13)	C10—C11—H11C	109.5
C13—P2—Ni1	115.87 (12)	H11A—C11—H11C	109.5
F6—P3—F5	91.51 (14)	H11B—C11—H11C	109.5
F6—P3—F7	90.39 (14)	C10—C12—H12A	109.5
F5—P3—F7	90.82 (16)	C10—C12—H12B	109.5
F6—P3—F4	91.55 (13)	H12A—C12—H12B	109.5
F5—P3—F4	90.44 (14)	C10—C12—H12C	109.5
F7—P3—F4	177.66 (15)	H12A—C12—H12C	109.5
F6—P3—F3	90.24 (13)	H12B—C12—H12C	109.5
F5—P3—F3	178.25 (14)	C14—C13—C15	111.4 (4)
F7—P3—F3	89.28 (14)	C14—C13—P2	109.7 (3)
F4—P3—F3	89.40 (13)	C15—C13—P2	113.2 (3)
F6—P3—F2	179.49 (15)	C14—C13—H13A	107.4
F5—P3—F2	88.98 (14)	C15—C13—H13A	107.4
F7—P3—F2	89.46 (13)	P2—C13—H13A	107.4
F4—P3—F2	88.59 (12)	C13—C14—H14A	109.5
F3—P3—F2	89.27 (13)	C13—C14—H14B	109.5
C2—O1—P1	112.2 (2)	H14A—C14—H14B	109.5
C6—O2—P2	111.79 (19)	C13—C14—H14C	109.5
C21—N1—N2	104.2 (3)	H14A—C14—H14C	109.5
C21—N1—Ni1	129.7 (2)	H14B—C14—H14C	109.5
N2—N1—Ni1	126.1 (2)	C13—C15—H15A	109.5
C19—N2—N1	111.9 (3)	C13—C15—H15B	109.5
C19—N2—H2A	124.1	H15A—C15—H15B	109.5
N1—N2—H2A	124.1	C13—C15—H15C	109.5
C2—C1—C6	115.1 (3)	H15A—C15—H15C	109.5
C2—C1—Ni1	122.9 (2)	H15B—C15—H15C	109.5
C6—C1—Ni1	121.9 (3)	C17—C16—C18	111.5 (4)
C3—C2—C1	124.4 (3)	C17—C16—P2	111.9 (3)
C3—C2—O1	118.4 (3)	C18—C16—P2	109.8 (3)
C1—C2—O1	117.2 (3)	C17—C16—H16A	107.9
C4—C3—C2	116.3 (3)	C18—C16—H16A	107.9
C4—C3—H3A	121.9	P2—C16—H16A	107.9
C2—C3—H3A	121.9	C16—C17—H17A	109.5
F1—C4—C5	118.0 (3)	C16—C17—H17B	109.5
F1—C4—C3	117.9 (4)	H17A—C17—H17B	109.5
C5—C4—C3	124.0 (3)	C16—C17—H17C	109.5
C4—C5—C6	116.4 (3)	H17A—C17—H17C	109.5
C4—C5—H5A	121.8	H17B—C17—H17C	109.5
C6—C5—H5A	121.8	C16—C18—H18A	109.5
C5—C6—O2	118.1 (3)	C16—C18—H18B	109.5
C5—C6—C1	123.7 (3)	H18A—C18—H18B	109.5
O2—C6—C1	118.1 (3)	C16—C18—H18C	109.5
C8—C7—C9	111.6 (3)	H18A—C18—H18C	109.5
C8—C7—P1	107.9 (2)	H18B—C18—H18C	109.5
C9—C7—P1	113.3 (3)	N2—C19—C20	107.1 (3)
C8—C7—H7A	107.9	N2—C19—H19A	126.4
C9—C7—H7A	107.9	C20—C19—H19A	126.4
P1—C7—H7A	107.9	C19—C20—C21	105.7 (3)
C7—C8—H8A	109.5	C19—C20—H20A	127.1
C7—C8—H8B	109.5	C21—C20—H20A	127.1
H8A—C8—H8B	109.5	N1—C21—C20	111.1 (3)
C7—C8—H8C	109.5	N1—C21—H21A	124.5
H8A—C8—H8C	109.5	C20—C21—H21A	124.5
